# Expansion of IgG+ B-Cells during Mitogen Stimulation for Memory B-Cell ELISpot Analysis Is Influenced by Size and Composition of the B-Cell Pool

**DOI:** 10.1371/journal.pone.0102885

**Published:** 2014-07-22

**Authors:** Anja Scholzen, Wiebke Nahrendorf, Jean Langhorne, Robert W. Sauerwein

**Affiliations:** 1 Department of Medical Microbiology, Radboud university medical center, Nijmegen, The Netherlands; 2 Division of Parasitology, MRC National Institute for Medical Research, London, United Kingdom; University of Copenhagen and Rigshospitalet, Copenhagen, Denmark

## Abstract

The memory B-cell (MBC) ELISpot assay is the main technique used to measure antigen-specific MBCs as a readout of humoral immune memory. This assay relies on the ability of MBCs to differentiate into antibody-secreting cells (ASC) upon polyclonal stimulation. The total number of IgG+ ASCs generated by mitogen-stimulation is often used as a reference point; alternatively antigen-specific MBCs are expressed as a frequency of post-culture peripheral blood mononuclear cells (PBMC) as a surrogate for absolute frequencies. Therefore, it is important to know whether IgG+ B-cells are uniformly expanded during the preceding mitogen-culture as a true reflection of MBC frequencies *ex vivo*. We systematically compared B-cell phenotype and proportions before and after mitogen stimulation in cultures of 269 peripheral blood mononuclear cell samples from 62 volunteers by flow cytometry and analyzed the number of resulting ASCs. Our data show that the number of total IgG+ ASCs detected by ELISpot after mitogen stimulation correlates with the proportion of IgG+ MBCs *ex vivo*, highlighting its general robustness for comparisons of study cohorts at group level. The expansion of total and IgG+ B-cells during mitogen-stimulation, however, was not identical in all cultures, but influenced by size and composition of the *ex vivo* B-cell compartment. The uncorrected readout of antigen-specific MBCs per million post-culture PBMCs therefore only preserves the quality, but not the magnitude of differences in the *ex vivo* MBC response between groups or time points, particularly when comparing samples where the B-cell compartment substantially differs between cohorts or over time. Therefore, expressing antigen-specific cells per total IgG+ ASCs is currently the best measure to correct for mitogen-culture effects. Additionally, baseline information on the size and composition of the *ex vivo* B-cell compartment should be supplied to additionally inform about differences or changes in the size and composition of the *ex vivo* MBC compartment.

## Introduction

Humoral immunity is crucial to combat many infections and to provide protection against re-infection and after vaccination. Primarily, antibodies are used as readouts for humoral immunity since they can be easily measured by enzyme-linked immunoabsorbance assay (ELISA). Long-term humoral immune memory is, however, not only conveyed by antibody-producing long-lived plasma cells, but also relies on the efficient acquisition and maintenance of memory B-cells (MBCs), who upon antigen re-encounter can rapidly develop into antibody secreting cells (ASCs) to mount a strong secondary antibody response [1].

Circulating MBCs have low frequencies and are quiescent, i.e. do not secrete antibody. Two main methods addressing these challenges have been developed to quantify the magnitude of the circulating MBC response: Direct *ex vivo* quantification can be performed using flow cytometry upon labelling of MBCs with fluorescently labelled monomeric or tetrameric antigens [2]–[4]. Alternatively, MBC-secreted antibodies can be quantified (by Enzyme-linked ImmunoSpot assay (ELISpot) or ELISA) following a pre-activation step using mitogens to differentiate MBCs into ASCs [5]. This method is readily applicable to large numbers of samples and antigens (provided sufficient cells are available from each sample), without the need for fluorescent labelling, which can be challenging for individual antigens [6]. It has thus become the main readout used to measure antigen-specific MBCs in the context of infections, vaccinations or allergy [6]–[12]. A necessary underlying, but yet untested, assumption made when using the MBC ELISpot to quantify antigen-specific responses is that MBCs are differentiated into ASCs at a fixed ratio [13]. Antigen-specific MBC responses measured using this technique are either reported as the number of ASC per million post-culture peripheral blood mononuclear cells (PBMC), or as percentage of total ASCs. Expressing antigen-specific cells as a proportion of ASC-differentiated MBC corrects for variation in both total MBC precursor frequencies and potential differences in expansion during mitogen culture between donors. Reporting antigen-specific ASCs as per million post-culture PBMCs is mostly used to get insights into the absolute frequency of antigen-specific MBCs. This is done to also take into account inter-individual variations in total MBC frequencies particularly when comparing across age groups [11], [13] that differ not just in their antigen-experience but also the size and composition of the (memory) B-cell compartment [11], [14]. This readout, however, does not correct for expansion and potential skewing of the MBC compartment during mitogen culture. It is therefore important to know (i) whether B-cells are indeed consistently expanded during mitogen culture, (ii) which factors influence this expansion, and (iii) whether the total number of Ig-secreting cells generated truly reflects MBC frequencies *ex vivo*. One study has addressed this last point for antigen-specific MBCs [4], but the readout by ELISA did not allow comparisons of ASCs following mitogen stimulation and *ex vivo* MBCs on the single-cell level. A follow-up study demonstrated that the two possible readouts after culture with mitogens, limiting dilution ELISpot/ELISA and bulk culture ELISpot, correlated for two out of the three antigens analyzed [6], supporting the use of the cheaper and less time consuming bulk ELISpot. However, none of these studies investigated the effect of mitogen culture on B-cell expansion, which would have affected both post-culture read-outs equally.

In this study, we therefore investigated both the consistency of B-cell expansion during mitogen culture as well as the robustness of this assay to detect total IgG+ ASCs that correspond to *ex vivo* IgG+ MBC frequencies, by systematically comparing B-cell phenotype and proportions before and after mitogen stimulation in a large number of human PBMC cultures.

## Materials and Methods

### Peripheral blood mononuclear cells

To enable systemic analysis of B-cell expansion and ASC generation in a large number of mitogen-stimulation cultures, we took advantage of 269 individual PBMC samples that were collected longitudinally from 62 healthy adult volunteers (age range 18–32 years) enrolled in two clinical trials conducted at the Radboud university medical center (Nijmegen, The Netherlands) and the Leiden University Medical Centre (Leiden, The Netherlands) between March 2011 and April 2012. Both studies received approval by the Central Committee for Research Involving Human Subjects of The Netherlands (CCMO; NL34273.091.10 and NL33904.091.10) and volunteers enrolled in the studies provided written informed consent. The study team complied with the Declaration of Helsinki and Good Clinical Practice including monitoring of data. The trials are registered at ClinicalTrials.gov, number NCT01236612 and NCT01218893.

In Study A [15], volunteers received a prophylactic dose of chloroquine for three months and bites from 3×15 *Plasmodium falciparum* (*Pf*)-infected mosquitoes (Chloroquine prophylaxis and sporozoites, CPS), and were subjected to challenge with *Pf*-infected red blood cells (n = 9) or infective mosquito bites (n = 5). Ten volunteers received only chloroquine prophylaxis and then a single *Pf* challenge either with *Pf*-infected red blood cells (n = 5) or mosquito-bites (n = 5). In Study B [16] volunteers undergoing chloroquine chemoprophylaxis were immunized with bites from either three times 15 (n = 5), ten (n = 9) or five (n = 10) *Pf*-infected mosquitoes, or not-immunized (n = 5). All immunized and control subjects in this study were subjected to mosquito-bite challenge.

Sampling of citrate anti-coagulated peripheral blood for immunological analysis was performed at different time points using CPT vacutainers (Becton Dickinson). In Study A and B, samples were available for each volunteer from D0 (pre-immunization, before onset of chloroquine prophylaxis), day before challenge (C−1) and 35 days after challenge (C+35). In Study B, additional samples were available from one month (28 days) after each of the three immunizations (for CPS-immunized volunteers only) as well as 140 days after challenge (C+140). Additionally, we included single samples from 9 healthy, Dutch adult malaria-naïve volunteers. At none of the blood collection time points for this study was any of the volunteers infected with *Pf*. PBMCs were isolated by density centrifugation. Following four washes in phosphate buffered saline (PBS), PBMCs were cryopreserved at 20×10^6^ cells/ml in ice-cold fetal calf serum (FCS; Gibco)/10% DMSO (Merck) using Mr. Frosty freezing containers (Nalgene). PBMC samples were stored in vapour phase nitrogen.

### Mitogen stimulation

Differentiation of MBCs into ASCs in was initiated in bulk PBMC cultures based on a previously established protocol [5], using a stimulation cocktail composed of pokeweed mitogen (PWM), *S. aureus* Cowan I protein A (SAC) and CpG. IL-10 was added to the stimulation mix since a previous study showed that this enhanced the efficiency of MBC into ASC differentiation by more than 9 fold [6]. Briefly, PBMCs were thawed for 30 sec in a 37°C water bath and cold RPMI medium was immediately added drop wise. After washing, the cells were re-suspended in RPMI containing 10% FCS, 100 U/ml penicillin/streptomycin, 100 mM HEPES, 50 mM 2-β-Mercaptoethanol and 2 mM L-Glutamine (all Invitrogen) and counted. 1×10^6^ cells/ml were added to 25 cm^2^ cell culture flasks (Greiner). Culture medium was supplemented with 50 ng/ml PWM lectin derived from *Phytolacca americana* (Sigma-Aldrich), 1∶5000 Protein A from *Staphylococcus aureus*, Cowan Strain (Sigma-Aldrich), 2.5 µg/ml ODN 2006 (Type B CpG nucleotide-human TLR9 ligand; InvivoGen tlrl-2006) and 25 ng/ml recombinant human IL-10 (PeproTech) and incubated at 37°C, 5% CO_2_ for 5 days.

### Memory B-cell ELISpot

MultiScreen Filter PVDF Immobilon plates (MSIPS4510, Millipore) were pre-wetted with 35% Ethanol, rinsed twice with PBS and coated with 10 µg/ml monoclonal antibodies to human IgG (clones MT91/145; Mabtech) diluted in phosphate buffered saline (PBS). After overnight incubation at 4°C, plates were washed (1× PBS/0.05% Tween 20 (Sigma-Aldrich, PBST), 3× PBS) and blocked with 1% bovine serum albumin (BSA; Sigma-Aldrich) in RPMI for 2 h at 37°C. Cells were harvested from the flasks, counted and seeded in quadruplicates at both 1200 and 4000 cells per well. Filter plates were incubated for 6 h at 37°C, 5% CO_2_ with minimized ambient vibration. After washing (4× PBS, 4× PBST), immobilized IgG was labeled using polyclonal goat anti-human IgG (Fcγ) alkaline phosphatase (1∶1000 in PBST containing 0.5% FCS; Mabtech) overnight at 4°C. Following thorough washing (4× PBST, 4× PBS, 3× distilled water), alkaline phosphatase substrate BCIP/NBT (Mabtech) was added and the filter plates were kept in the dark until fully developed. Developed plates were rinsed with water, left to dry and stored protected from light until reading using the CTL ImmunoSpot Reader (Cellular Technology Ltd.). The mean number of IgG+ ASCs in quadruplicate wells was calculated per million PBMC and averaged between the two cell concentrations. To check for assay background some wells on every plate received only medium instead of cells (no background detected) or 4×10^5^ cells were added to wells coated with 5 µg/ml the irrelevant antigen Bovine Serum Albumin (background: 0.33 spots/well).

### Flow cytometry analysis

IgG+ B-cells and B-cell proportions were analyzed prior to (*ex vivo*; stain 1; **Figure S1**) and after mitogen culture (stain 2; **Figure S2**). B-cell subsets *ex vivo* were distinguished as described previously [17]. For post-mitogen culture phenotyping we chose a different staining panel for the following reasons: (i) mitogen stimulation results in a down-regulation of CD19 expression, necessitating the inclusion of CD20 to identify B-cells. (ii) we aimed to assess proliferation after stimulation, thus including Ki67. (iii) in previous experiments we found that CD21 as well as IgD expression are down-regulated during culture of B-cells, while CD38 expression is strongly up-regulated in the majority of B-cells by mitogen-stimulation (**Figure S3**), thus preventing us to use the same gating strategy to distinguish different B-cells subsets after 5 days of culture.

5×10^5^ to 1×10^6^ cells per stain were transferred to 96 wells V-bottom plate (Sarstedt), washed with PBS and incubated for 30 min on ice with 50 µl viability dye diluted in PBS (fixable viability dye eFluor 450, eBioscience (stain 1); aqua LIVE/DEAD fixable dead cell stain, Invitrogen (stain 2)). Cells were washed twice with staining buffer (PBS/0.5% BSA), and stained with 50 µl antibody cocktail diluted in staining buffer for 30 min at room temperature (RT). Antibodies used for surface staining are listed in **Table 1** **and** **Table 2**. After another wash, cells were re-suspended in 50 µl fixation/permeabilization buffer (eBioscience), incubated for 30 min at 4°C, and washed with 150 µl permeabilization buffer (eBioscience). For intracellular staining, cells were incubated for 30 min at RT with 50 µl antibody cocktail (stain 2; Ki67 AlexaFluor 647 (B56); BD Biosciences) diluted in permeabilization buffer (eBioscience). Cells were washed with permeabilization buffer and re-suspended in 200 µl PBS/1% paraformaldehyde. 100,000 to 200,000 events per sample were acquired on a Cyan ADP 9-colour flow cytometer (Dako/Beckman Coulter) and analysis performed using FlowJo v9.2 software.

**Table 1:** Antibodies used for *ex vivo* flow cytometry analysis.

Purpose	Target antigen	Fluorochrome	Clone	Supplier
**Viability**	fixable viability	eFluor 450	n/a	eBioscience
**Dump channel** a	CD3	FITC	OKT3	Biolegend
	CD56	FITC	HCD56	Biolegend
	CD14	FITC	HCD14	Biolegend
**B-cell lineage**	CD19	APC-eF780	HIB19	eBioscience
**B-cell subset**	CD10	ECD	ALB1	BeckmanCoulter
	CD38	PerCp	HIT2	Biolegend
	CD27	PC7	IA4CD27	BeckmanCoulter
	CD21	APC	B-ly4	BD Biosciences
	IgD	biotin	IA6-2	BD Biosciences
	Streptavidin	Pacific Orange	n/a	Invitrogen
**BCR isotype**	IgG	PE	IS11-3B2.2.3	Miltenyi Biotech

a Dump channel comprised of lineage markers to gate out non-relevant PBMC subsets.

n/a = not applicable.

BCR = B-cell receptor.

**Table 2:** Antibodies used for post-culture flow cytometry analysis.

Purpose	Target antigen	Fluorochrome	Clone	Supplier
**Viability**	lived/dead stain	aqua	n/a	Invitrogen
**Dump channel** a	CD3	FITC	OKT3	Biolegend
	CD56	FITC	HCD56	Biolegend
	CD14	FITC	HCD14	Biolegend
**B-cell lineage**	CD19	APC-eF780	HIB19	eBioscience
	CD20	Pacific Blue	2H7	Biolegend
**B-cell activation**	CD38	PerCp	HIT2	Biolegend
**BCR isotype**	IgG	PE	IS11-3B2.2.3	Miltenyi Biotech
**Proliferation**	Ki67	Alexa Fluor 647	B56	BD Biosciences

a Dump channel comprised of lineage markers to gate out non-relevant PBMC subsets.

n/a = not applicable.

BCR = B-cell receptor.

### Statistical analysis

Data were analyzed using GraphPad Prism v5. B-cell proportions *ex vivo* versus post-culture were compared by Wilcoxon matched pairs signed rank test. Skewing of distributions was tested by D'Agostino and Pearson omnibus normality test. Relationships between B-cell proportions, expansion (fold change; calculated by dividing for each sample the absolute number of total (or IgG+) B-cells post mitogen-culture by the absolute number of total (or IgG+) B-cells *ex vivo*) and ASCs were analyzed by non-parametric Spearman correlation. Correlation coefficients listed are for PBMC samples of the 62 volunteers at baseline, i.e. a time point when all of them where malaria-naïve and none of them was undergoing chloroquine prophylaxis. Correlation coefficients obtained for baseline samples only were comparable to those obtained when analyzing all 269 cultures from all collected time points.

## Results

**Figure 1:**
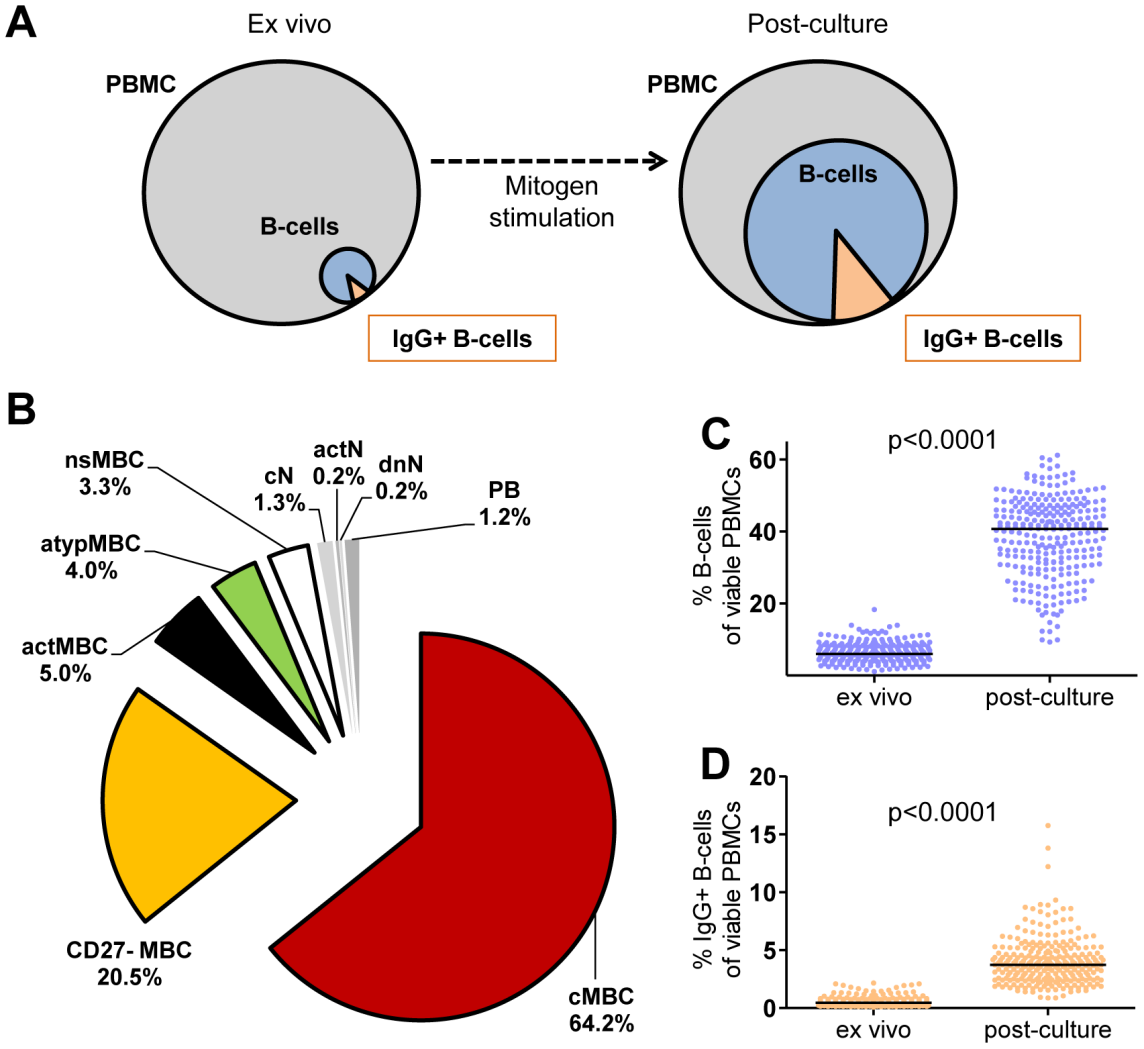
Expansion of total and IgG+ B-cells following mitogen-stimulation. Panel (A) schematically illustrates the cellular composition of PBMCs directly *ex vivo* and after mitogen culture. The grey circle represents total PBMCs, the blue circle all CD19+ B-cells and the orange triangle IgG+ B-cells. (B) The composition of the IgG+ B-cell compartment *ex vivo* was analyzed by flow cytometry and is depicted as median proportions of individual B-cell subsets within total CD19+ B-cells for baseline samples of 62 donors. The individual B-cell subsets were subdivided based on IgD, CD38, CD10, CD21 and CD27 expression and include five memory B-cell subsets: classical MBCs (cMBCs, red), CD27− MBCs (yellow), activated MBCs (actMBC, black), atypical MBCs (atypMBC, green) and non-switched MBCs (nsMBC, white). Depicted in shades of grey are plasma blasts (PB), activated naïve B-cells (actN), classical naïve B-cells (cN) and double-negative naïve B-cells (dnN). Panels C–D show *ex vivo* and post-culture proportions of total CD19+ B-cells (C, blue dots) and IgG+CD19+ B-cells (D, orange dots) within viable PBMCs. Black lines indicate the median. Dots show all 269 cultures.

Applying both flow cytometry and ELISpot, we conducted a systematic large-scale analysis of 269 PBMC samples (derived from 62 donors sampled over 1–7 time points) before and after mitogen-stimulation (**Figure 1A**). We firstly determined the *ex vivo* composition of the B-cell compartment (for subset definitions see **Table 3** **and Figure S1**), and particularly of IgG+ MBCs, from which IgG+ ASCs originate. Upon thawing of cryopreserved PBMCs, B-cells made up 5.97% (median, interquartile range (IQR) 4.26–8.25%) of PBMCs, and 7.86% (median; IQR 5.69–11.5%) of these B-cells were IgG+. The IgG+ B-cell compartment was largely composed of CD38lowIgD-CD10− MBCs (**Figure 1B**), dominated by CD21+CD27+ classical MBCs (cMBC; median 63.4%, IQR 55.9–67.8%), followed by CD21+CD27− MBCs (median 20.3%, IQR 15.4–24.6%). Three other MBC populations constituted 3.3–5% each of the IgG+ B-cell compartment, i.e. CD21−CD27− atypical MBCs (atypMBC), CD21−CD27+ activated MBC (actMBC) and IgD+CD21+CD27+ non-switched MBCs (nsMBC). With the exception of a slight dip in total B-cells four weeks after the last immunization, both the size of the total and IgG+ circulating B-cell compartment, and the proportions of the different MBC subsets remained constant over time in the 53 donors from which multiple samples were available (**Table S1 and Table S2**), highlighting that the interventions the volunteers underwent in the clinical trials had no biasing effect on the samples analyzed.

**Table 3:** Definition of B-cell subsets *ex vivo*.

B-cell subset	Abbreviation	IgD	CD38	CD10	CD27	CD21
**Classical MBCs**	**cMBC**	−	low	−	+	+
**CD27− MBCs**	**CD27− MBC**	−	low	−	−	+
**Activated MBCs**	**actMBC**	−	low	−	+	−
**Atypical MBCs**	**atypMBC**	−	low	−	−	−
**Non-switched MBCs**	**nsMBC**	+	low	−	+	+
**Classical naïve B-cells**	**cN**	+	low	−	−	+
**Activate naïve B-cells**	**actN**	+	low	−	+	−
**Double-negative naïve B-cells**	**dnN**	+	low	−	−	−
**Transitional B-cells**	**TBC**	+	hi	+	−	n/a
**Plasma blasts**	**PB**	−	hi	−	+	n/a

Recovery of total seeded PBMC after 5 days of culture in the presence of mitogens was 95.45% (median, IQR 82.9–112.1%). Consistent with 90.4% (median; IQR 87.5–92.1%) of B-cells proliferating at day 5 (based on Ki67 staining, **Figure S3**), mitogen-stimulation successfully increased the proportion of total B-cells (**Figure 1C**, p<0.0001) and IgG+ B-cells within recovered PBMCs in all cultures (**Figure 1D**, p<0.0001).

**Figure 2:**
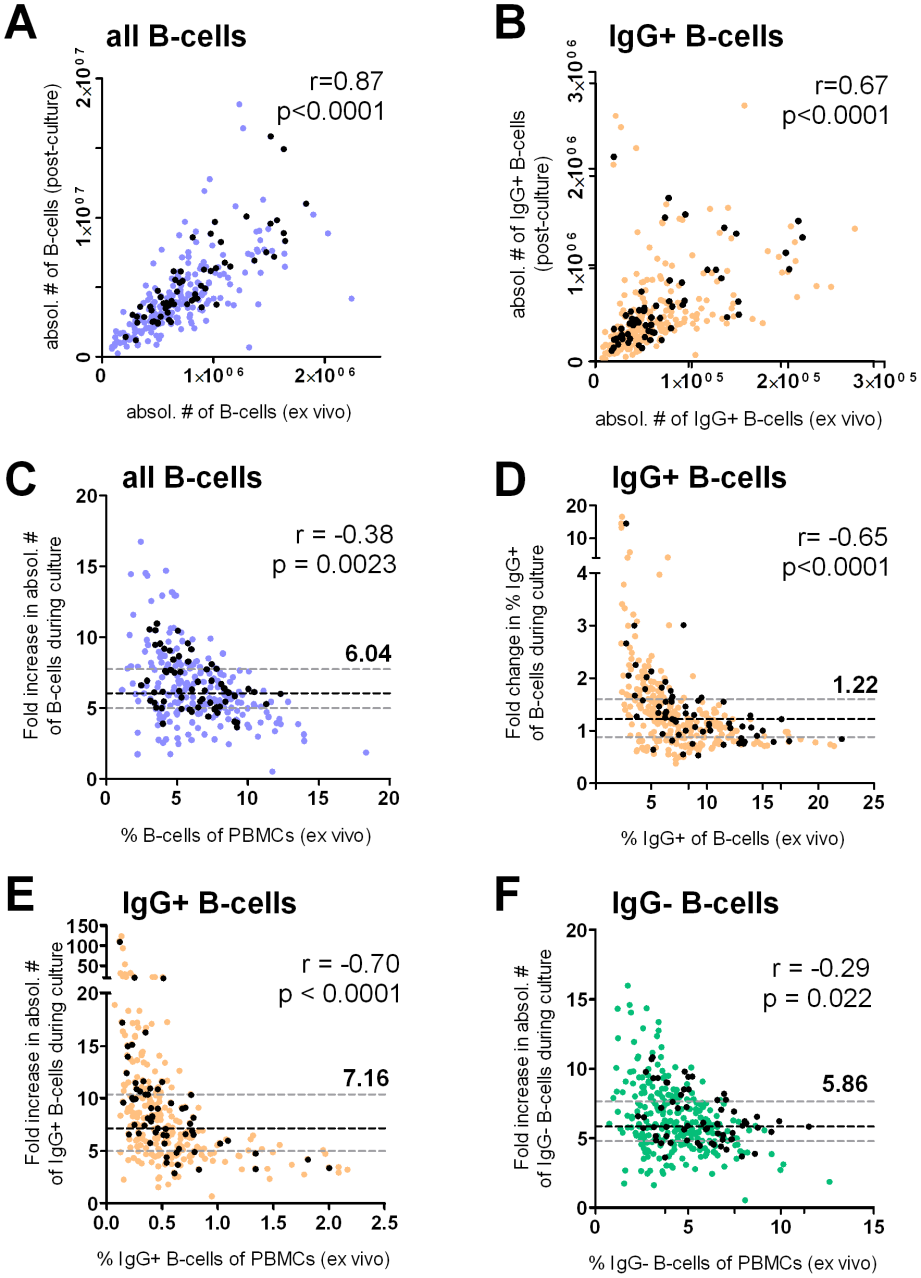
Size and composition of the B-cell compartment *ex vivo* influences B-cell expansion during culture. Flow cytometry analysis was performed to determine proportions and subsequently calculate absolute numbers of (A) total CD19+ B-cells (blue dots) and (B) IgG+CD19+ B-cells (orange dots) *ex vivo* and post-culture. *Ex vivo* proportions of (C) total CD19+ B-cells (blue dots) within viable PBMCs were plotted against the fold change in their absolute numbers, and (D) IgG+ B-cells (orange dots) within total CD19+ B-cells were plotted against the fold change in their proportion within the B-cell compartment. (E) Proportions of IgG+CD19+ B-cells (orange dots) and (F) IgG−CD19+ B-cells (green dots) within viable PBMCs were plotted against the fold increase in their respective absolute numbers post-culture compared to *ex vivo*. Colored dots show cultures from all 269 stimulated samples (3–7 time points per volunteer), while black dots show the cultures from only the 62 baseline samples (1 for each individual volunteer). The black dashed line indicates the median fold change (with value), grey dotted lines represent the upper and lower limit of the interquartile range. Spearman r and p values are shown for analysis of baseline samples (black dots) from the 62 donors assessed.

While the absolute number of both total and IgG+ B-cells prior to culture correlated with their number after culture, this relationship was stronger for total B-cells (Spearman r = 0.87, p<0.0001; **Figure 2A**), than for IgG+ B-cells (r = 0.67, p<0.0001; **Figure 2B**). For total B-cells, the median fold increase in absolute numbers over 5 days of culture was 6.04 (IQR 5.0–7.74; p<0.001), and this increase in total B-cell numbers during mitogen culture was not normally distributed (D'Agostino and Pearson omnibus normality test; skewness 1.06, kurtosis 2.04). Since all mitogen cultures were performed under identical conditions, the most likely confounding factor was the variation in the starting proportion of total B-cells (median 5.97%, range 1.1–18.3%). Indeed, we found that expansion of B-cells during mitogen culture was variable and dependent of B-cell proportions before stimulation with an inverse and non-linear (exponential decay) relationship between the *ex vivo* proportion and the fold increase in both their percentage (r = −0.69, p<0.0001, data not shown) and absolute number (r = −0.38, p = 0.023; **Figure 2C**) during culture. The largest expansion was found in cultures with the lowest starting proportions of total B-cells.

We next addressed whether mitogen culture affected the composition of the B-cell compartment. Overall, proportions of IgG+ cells within the B-cell pool only increased slightly (pre- versus post-culture: median with IQR 7.68% (5.7–11.5%) and 10.3% (7.7–12.8%), respectively). However, as for B-cells within total PBMCs, we also found that the *ex vivo* percentage of IgG+ cells strongly influenced their expansion within the B-cell pool, with a strong inverse and skewed correlation (**Figure 2D**, r = −0.65, p<0.0001) between their starting proportions and fold increase during culture (median fold change post-culture/*ex vivo* 1.22, IQR 0.88–1.6; skewness 7.1; kurtosis 58.1). These effects of pre-culture size and composition of the B-cell pool on the efficiency of mitogen-driven expansion also affected the increase in IgG+ B-cells, with a strong inverse correlation between starting proportions and the increase in their percentage (r = −0.76, p<0.0001, data not shown) or absolute numbers (r = −0.70, p<0.0001; **Figure 2E**): This was particularly evident in cultures with a low proportion of starting IgG+ B-cells (<0.5% of PBMCs; 155/269), where expansion was higher (median fold increase 9.3), more variable (IQR 6.7–13.4) and skewed (skewness 5.6, kurtosis 34.7) than in cultures with higher pre-culture proportions of IgG+ B-cells (≥0.5% of PBMC; 114/269; median fold increase 5.3; IQR 4.4–6.7; skewness 2.2, kurtosis 7.8). Finally, expansion of IgG+ B-cells was higher than that of IgG− B-cells (**Figure 2E, F**; median fold increase 7.16 vs 5.86; p<0,0001), and expansion of IgG+ B-cells more strongly influenced by their starting proportion (skewness 6.9, kurtosis 55.9; Spearman r = −0.70) than IgG− B-cells (skewness 0.99, kurtosis 1.7; Spearman r = −0.29). Taken together, differences in both size and composition of the *ex vivo* B-cell compartment do directly influence the outcome of mitogen-stimulation cultures.

**Figure 3:**
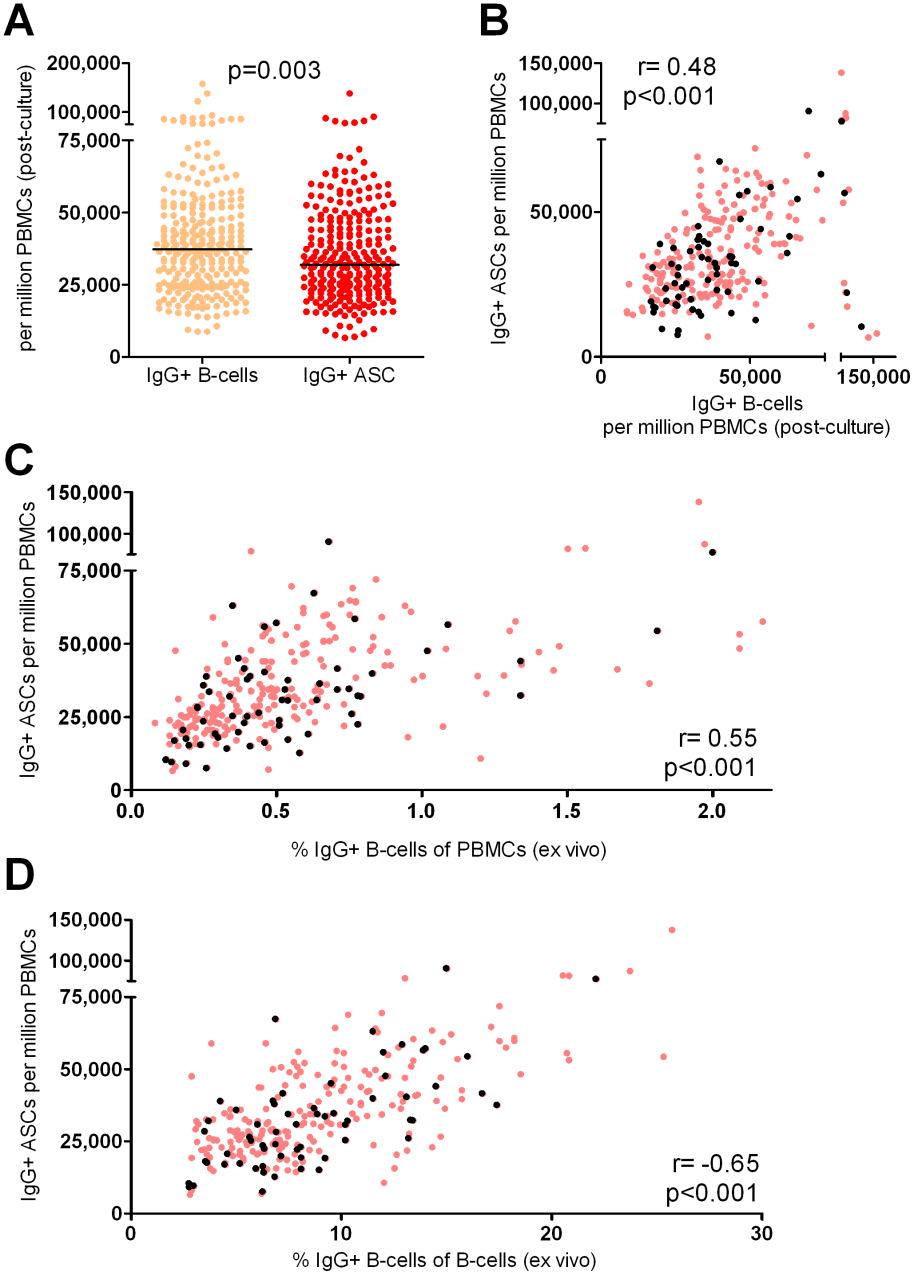
Relationship between post-culture IgG+ ASC numbers and *ex vivo* or post-culture IgG+ B-cell proportions. After 5 days of mitogen culture, proportions of IgG+ B-cells were analyzed by flow cytometry (orange dots) and IgG+ ASCs were quantified by ELISpot (red dots) and expressed as per million post-culture PBMCs (A). Black lines indicate the median. Post-culture (B) and *ex vivo* proportions (C) of IgG+ cells within PBMCs, and *ex vivo* proportions of IgG+ cells within CD19+ B-cells (D) were plotted against the number of IgG+ ASCs per million PBMCs. Light red dots show cultures from all 269 stimulated samples (3–7 time points per volunteer), while black dots show the cultures from only the 62 baseline samples (1 for each individual volunteer).

When comparing the number of IgG+ B-cells determined by flow cytometry to the number of IgG+ ACSs detected by ELISpot, both per million PBMCs post-culture, we found that the number of IgG+ B-cells, although in a similar range, was slightly but significantly higher than that of IgG+ ASCs (**Figure 3A**; p = 0.003). This suggests that the majority of IgG+ B-cells expanded during mitogen culture also differentiate into ASCs. There was, however, only a relatively weak correlation between the proportion of IgG+ B-cells and IgG+ ASCs after mitogen culture (Spearman r = 0.48, p<0.0001; **Figure 3B**), showing that for a large number of cultures, staining of post-culture PBMCs for surface IgG will overestimate the potential of these cells to secrete IgG, as detected by ELISpot. When IgG+ ASC numbers (per million PBMCs) were compared instead to the *ex vivo* proportion of IgG+ cells within PBMCs prior to culture, this correlation improved (r = 0.55, p<0.0001; **Figure 3C**), showing that IgG+ ASCs detected by MBC ELISpot are overall a good estimate of *ex vivo* MBC frequencies. Moreover, when taking out variations in the size of the B-cell pool by analyzing *ex vivo* IgG+ proportions within the B-cell compartment, the correlation with IgG+ ASC numbers post-mitogen culture improved further (r = 0.65, p<0.0001; **Figure 3D**). This correlation was the same for IgG+ cMBCs, which constitute the largest proportion of the IgG+ B-cell pool (r = 0.65, p<0.0001). IgG+ ASC numbers post-culture further correlated with the *ex vivo* proportions of IgG+ CD27− MBCs (r = 0.51, p<0.0001), the second largest population within IgG+ MBCs, but not for IgG+ atypMBCs (r = 0.24, p = 0.06). Of note, across all baseline samples of 62 volunteers, the ratio of atypMBCs/cMBCs correlated inversely with the proportion of IgG+ cMBCs *ex vivo* (r = −0.54, p<0.0001), but correlated positively with the expansion of IgG+ B-cell numbers during mitogen culture (r = 0.49, p<0.0001, data not shown).

## Discussion

The MBC ELISpot assay is the most widely used method to quantify MBC responses in addition to plasma antibodies as an independent readout of humoral immune memory [6]–[12], [18]. A basic assumption when using the MBC ELISpot assay to estimate the frequency of antigen-specific MBCs is that in the preceding mitogen-culture, all MBCs are expanded and differentiate into ASCs in a constant manner or at a fixed ratio, although this has not been examined to date [13]. We show here that the expansion of total and IgG+ B-cells during culture is not constant, but instead influenced by both the size and composition of the *ex vivo* B-cell compartment, which vary widely between individuals.

At group level, the number of IgG+ ASCs detected by ELISpot after mitogen stimulation indeed reflects the proportion of IgG+ MBCs *ex vivo*, in particular for the two major populations of IgG+ MBCs, i.e. cMBCs and CD27− MBCs. This good correlation between total input (*ex vivo* IgG+ MBC) and output (IgG+ ASC) is in line with a previous small-scale study on TTX-specific MBCs [4] and highlights the general robustness of this assay to compare antigen-specific MBC frequencies on group level (e.g. between different cohorts). This relationship was found across cultures from 62 volunteers with a wide range of *ex vivo* IgG+ MBC frequencies. We also observed, however, that the expansion of both B-cells and IgG+ B-cells during culture showed an inverse correlation with the size and composition of the pre-culture B-cell compartment. This was particularly evident for IgG+ MBCs. As a result, when total or antigen-specific MBCs are expressed as a proportion of total post-mitogen culture PBMCs to estimate their frequency, this introduced bias into the readout, rather than allowing to take into account inter-individual variations in total *ex vivo* MBC frequencies [13].

The reason underlying this inverse relation between the *ex vivo* proportion of total or IgG+ B-cells with their respective increase in absolute numbers during culture remains unknown. One possibility is that the T-cell compartment is relatively larger in cultures with lower B-cell proportions, and thus more T-cells available for cytokine production upon PWM-stimulation, providing more favorable expansion conditions than in cultures with higher B-cell and lower T-cell numbers. This factor would influence total B-cell expansion whenever mitogen-stimulation is performed in bulk PBMC cultures. It does not, however, explain the skewing for IgG+ B-cells within the B-cell compartment, since cultures with the lowest total B-cell proportion where not those with the lowest proportion of IgG+ B-cells within the B-cell compartment and vise versa. Alternatively, assuming the same expansion efficiency in all samples, IgG+ B cells in samples with a high starting proportion might die in culture due to crowding, limited nutrients or toxin build up, whereas IgG+ B cells in samples with a low starting proportion can continue to proliferate and differentiate until the end of the 5 day culture. While this again may be true for the B-cell compartment as a whole, we consider it unlikely to be the sole reasons for the strong inverse relationship between starting-proportion and expansion of IgG+ B-cells: Firstly, the strong skewing effect was also observed within the B-cell compartment for IgG+ B-cells. Secondly, albeit 10 times more numerous, starting proportion of IgG− B-cells (containing mostly naïve B-cells and a few IgA+ or IgM+ MBCs) had only a very weak influence on their expansion in culture. This suggests that other reasons underlie these differences in expansion efficiency of IgG+ B-cells.

The skewing effect of B-cell frequency and composition particularly affected cultures with a low proportion of *ex vivo* IgG+ MBCs, which were expanded with a greater magnitude and variation than those in culture with high starting proportions. One potential explanation for this observation is that expansion of IgG+ MBCs might also be enhanced by direct cell-cell contact of IgG+ B-cells with PWM-activated T-cells (for instance via CD40-CD40L [19]): when the IgG+ B-cell population is smaller to begin with, the likelihood for direct cell-cell contact may be lower, and interactions thus more random, than when IgG+ B-cells are more frequent. In line with a greater variation in expansion, when *ex vivo* IgG+ MBC proportions were plotted against post-culture IgG+ ASCs, the spread was wider and the correlation coefficient weaker for cultures with lower starting proportions of IgG+ MBCs (less than 0.5% of starting PBMCs). Thus, special care should be taken when comparing absolute IgG+ MBC numbers between subjects particularly with low starting frequencies of IgG+ MBCs, such as young children [11], [14]. In such a case, expressing antigen-specific MBCs as a proportion of post-culture PBMCs gives even less insight into their actual *ex vivo* frequencies than when comparing across samples with a wide range of starting IgG+ MBC proportions. Expressing antigen-specific IgG+ MBCs as a proportion of IgG+ ASCs is therefore necessary to correct for this great variation in expansion. Prior knowledge of the starting concentration of IgG+ MBCs (e.g. by whole blood staining in a small volume prior to cryopreservation) might provide valuable additional information to accurately estimate differences in antigen-specific IgG+ MBC precursor frequencies: This information may be used to determine whether an actual difference in IgG+ MBC frequencies or MBC subsets exists between two groups, or whether they are comparable and differences in antigen-specific cells per total ASCs thus also reflect a different magnitude of the antigen-specific MBC response.

Another basic assumption of the MBC ELISpot assay is that all antigen-specific cell subsets have an equal ability to differentiate into ASCs. In our hands the number of post-culture surface IgG+ B-cells, however, did not fully predict the number of IgG+ ASCs. One potential reason could be the down-regulation of surface IgG expression on differentiating ASCs mitogen culture. This would lead to an underestimation of IgG+ ASCs, which could be rectified by performing intracellular IgG staining post culture. However, after mitogen culture the number of surface IgG+ B-cells slightly but significantly exceeds, and thus over- rather under-estimates the number of IgG+ ASCs. While we can further not formally show that all expanded IgG+ B-cells are indeed plasma blasts (since staining for CD27 was not performed in addition to CD38), we find that the number of post-culture IgG+ cells and IgG+ ASCs correlates (Spearman r = 0.48, p<0.0001), indicating that at least a major proportion of those cell populations does overlap. Moreover, these data indicate that not all IgG+ B-cells (*ex vivo* and post culture) actually acquire ASC function, as previously shown at the single cell level for isotype-switched MBCs after stimulation with CpG and cytokines [20], [21]. A marker identifying MBC subsets with the capacity to become ASCs is unfortunately still lacking [20], and this may further differ depending on the combination of stimuli chosen: Since the description of the MBC ELISpot by Crotty et al. [5], many different polyclonal stimulation protocols relying on the activation of Toll-like receptors as well as cytokine receptor signaling have been tested to effectively differentiate MBCs into ASCs. Here we choose a stimulation cocktail containing PWM, SAC, CpG and IL-10, which was previously shown to be the most efficient out of 12 different stimulation protocols tested [6]. Based on the data we provide, future studies using other effective stimulation protocols (e.g. IL-2 and R848 [9], [10], or IL-15 and CpG [7]) or purified B-cells [18] should determine whether the size and composition of the *ex vivo* B-cell pool influences the expansion of total and IgG+ B cells during culture.

Most IgG+ B-cells *ex vivo* are cMBCs, and their proportion correlated as strongly with IgG+ ASCs detected by ELISpot as the proportion of total *ex vivo* IgG+ B-cells. Curiously, low proportions of IgG+ cMBCs within the B-cell compartment *ex vivo* (and thus low numbers of IgG+ ASCs after mitogen culture) were predominantly found in volunteers with high *ex vivo* ratios of atypMBC to cMBC. This was particularly evident for one volunteer, who at all three time points, at which PBMCs were sampled, showed the highest number of IgG+ B-cells after mitogen stimulation but only very low IgG+ ASC numbers, suggesting that only a small fraction of the efficiently expanded IgG+ B-cells actually secreted IgG: this volunteer had a very low proportion of cMBCs and CD27− MBCs within IgG+ B-cells, but a particularly large proportion of atypMBCs prior to mitogen culture. This observation would be consistent with previous studies that reported atypMBCs from HIV-infected individuals to be less efficient in differentiating into IgG+ ASCs *in vitro*, and similar data are available for a very limited number of atypMBCs from malaria-exposed subjects [12], [22]. On the other hand, there is evidence that atypMBCs from malaria-exposed individuals may contribute to antibody production, since as for cMBCs, their BCR sequences can be matched to circulating plasma antibodies [23]. Additionally, bulk-sorted atypMBCs in this study contained both the membrane-bound and secreted form of IgG, however, a contamination of this population with non-atypMBC antibody-secreting cells cannot be fully excluded, since CD38hi cells were not depleted [23]. It therefore remains to be conclusively established whether atypMBCs indeed actively secrete antibodies *in vivo*, or simply differentiate into ASCs like cMBCs (just potentially less efficiently) and then contribute to IgG-secretion upon antigenic stimulation. The extent to which atypMBCs and cMBCs in both malaria-exposed and healthy, malaria-naïve individuals vary in their ability to differentiate into ASCs to different stimuli also remains to be established. Should this indeed be the case, then data not only on the size, but also the detailed composition of the *ex vivo* IgG+ MBC pool might be helpful for data interpretation, especially when comparing populations with a broad range of proportions of atypMBC, such as individuals of different age groups in malaria-endemic areas or in different transmission settings [11], [22].

Antigen-specific ASCs are often reported per million post-culture PBMCs to get insights into the frequency of circulating antigen-specific MBCs. This readout, however, does not correct for differential expansion and thus skewing of the MBC compartment during mitogen culture. Therefore only qualitative (higher or lower *ex vivo* frequencies of antigen-specific cells) but not quantitative differences between groups (which one aims to examine when using this readout) are preserved. This is specifically relevant for comparing antigen-specific MBCs between groups that markedly differ in size and composition of the B-cell compartment, such as between different age groups [11], [14]. A correction factor to accurately quantify antigen-specific MBC responses (taking into account both the size of the B-cell compartment and the proportion of IgG+ MBCs *ex vivo*), would simplify analysis, but its determination is complicated by two main aspects: (i) different IgG+ MBC subsets may have varying abilities to differentiate into ASCs and (ii) other stimulation protocols may introduce different degrees of skewing during culture, for instance since receptor expression varies between MBC subsets [24]. In the absence of such a correction factor, we conclude that it is not possible to accurately estimate MBC frequencies by MBC ELISpot on an individual level. For this question, limiting dilution assay or *ex vivo* staining with fluorescently-labeled antigens [3], [4] or B-cell tetramers [2] remain the methods of choice.

An immediate practical implication of our finding is thus the way that results from bulk-culture MBC ELISpot should be reported: Specifically, we recommend not to express antigen-specific MBCs as a proportion of post-culture PBMCs in an attempt to compare their frequencies - particularly between clearly different cohorts, since this actually introduces bias. Instead, to allow interpretation of MBC ELISpot data in a meaningful manner, we propose to always include total IgG+ ASCs as a reference point to correct for variations in MBC precursor frequencies and hence expansion during mitogen culture between donors. Between relatively homogenous groups (in regards to their B-cell compartment), expressing antigen-specific cells per total ASCs is currently the best measure to correct for mitogen-culture effects. The same is true for longitudinal studies where the size and composition of the (memory) B-cell compartment does not alter over time (as in the samples analyzed herein). In settings, however, where groups differ in the size and makeup of their (IgG+ memory) B-cell compartment (or these parameters alter over time in longitudinal studies) due to age or pathogen exposure, we propose that this information about the B-cell compartment should be additionally provided as a separate measure. This will also inform the reader appropriately whether it is simply the size of the MBC compartment that differs between (age) groups or alters over time, or the proportion of antigen-specific cells within the MBC compartment, or both – information that is lost when only providing the mitogen-culture biased readout of antigen-specific MBCs per post-culture PBMCs.

## Conclusions

The number of total IgG+ ASCs detected by ELISpot after mitogen stimulation correlates with the proportion of IgG+ MBCs *ex vivo*, highlighting the general robustness of this assay to compare MBC responses between different cohorts, i.e. at group level. The expansion of total and IgG+ B-cells during mitogen-stimulation, however, was not identical in all cultures, but influenced by both the size and composition of the *ex vivo* B-cell compartment, which vary widely between individuals. The uncorrected readout of antigen-specific MBCs per million post-culture PBMCs therefore only preserves the quality, but not the quantity of differences in the *ex vivo* MBC response between samples and thus groups or time points. Expressing antigen-specific cells per IgG+ ASCs is currently the best measure to correct for differences in the *ex vivo* B-cell compartment and resulting mitogen-culture effects. To be able to evaluate not only changes within the MBC compartment, but their actual magnitude within the circulation, additional information on the size and composition of the *ex vivo* B-cell compartment should be supplied as a separate measure, particularly under circumstances when the proportions IgG+ MBC are highly variable or proportions of B-cell subsets are altered by environmental factors either between groups or over time.

## Supporting Information

Figure S1 **Gating strategy of total and IgG+ B-cells** ***ex vivo*** **.** (A) CD19+ B-cell were identified following exclusion of debris, doublets, dead cells and CD3/CD56/CD14-positive cells. (B) shows gating for IgG+ B-cells. This IgG+ gate was later applied to individual B-cell subsets, which were gates as follows: (C) CD19+ B-cells were first subdivided based on IgD and CD38 expression. (D) CD10+ cells were excluded from all B-cell populations except IgD+CD38hi B-cells, which were specifically subgated based on CD10 expression. (E) CD38hi B-cells were divided into CD10−IgD−CD38hiCD27+ plasma blasts (PB, i) and CD10+ IgD+CD38hiCD27− transitional B-cells (TBC, ii). CD38lowCD10− B-cells were subdivided into four pairs of switched/memory (IgD−) and non-switched/naïve (IgD+) B-cell populations: CD21−CD27+ activated MBCs (actMBC, iii) and activated naïve B-cells (actN, iv); CD21+CD27+ classical MBCs (cMBC, v) and non-switched MBCs (nsMBC, vi); CD21+CD27− MBC (CD27− MBC, vii) and classical naïve B-cells (cN, viii); and CD21−CD27− atypical MBCs (atypMBC, ix) and double-negative naïve B-cells (dnN, x). (F) shows the proportions of the ten B-cell subsets of the total *ex vivo* B-cell pool and (G) the proportion of IgG+ cells within each individual B-cell subset for the baseline samples of all 62 volunteers.
(TIF)

Figure S2 **Gating strategy of total and IgG+ B-cells after 5 day mitogen culture.** Following (A) exclusion of debris, doublets and dead cells, B-cells were identified by (B) firstly gating on CD19+CD20+ cells and subsequently gating out CD3/CD56/CD14-positive cells (Boolean gating). (C) shows gating for IgG+ B-cells for two donors.
(TIF)

Figure S3 **CD38 and Ki67 expression on B-cells after 5 day mitogen culture.** (A) Ki67 and CD38 gates were based on negative populations in total post-culture PBMCs (after exclusion of debris, doublets and dead cells). (B) shows Ki67 and CD38 staining on post-culture B-cells. (C) Grey dots show the percentage of Ki67 and CD38 positive cells within post-culture B-cells cultures in all 269 stimulated samples, black lines indicate the median and error bars the interquartile range.
(TIF)

Table S1 B-cells, IgG+ B-cells and MBC subsets in Study A ex vivo samples over time. B-cell proportions and MBC subsets in PBMC samples from Study A volunteers (n = 14 CPS-immunized volunteers and n = 10 controls) were analyzed by flow cytometry. CD19+ B-cell were identified following exclusion of debris, doublets, dead cells and CD3/CD56/CD14-positive. Five MBC populations were identified in the CD38lowCD10− B-cells compartment as followed: IgD−CD21+CD27+ classical MBCs (cMBC), IgD−CD21+CD27− MBC (CD27− MBC), IgD−CD21−CD27+ activated MBCs (actMBC), IgD−CD21−CD27− atypical MBCs (atypMBC) and IgD+CD21+CD27+ non-switched MBCs (nsMBC).
(DOC)

Table S2 B-cells, IgG+ B-cells and MBC subsets in Study B ex vivo samples over time. B-cell proportions and MBC subsets in PBMC samples from Study B volunteers (n = 24 CPS-immunized volunteers and n = 5 controls) were analyzed by flow cytometry. CD19+ B-cell were identified following exclusion of debris, doublets, dead cells and CD3/CD56/CD14-positive. Five MBC populations were identified in the CD38lowCD10− B-cells compartment as followed: IgD−CD21+CD27+ classical MBCs (cMBC), IgD−CD21+CD27− MBC (CD27− MBC), IgD−CD21−CD27+ activated MBCs (actMBC), IgD−CD21−CD27− atypical MBCs (atypMBC) and IgD+CD21+CD27+ non-switched MBCs (nsMBC).
(DOC)
