# Supplementary figures and images for: Expansion of IgG+ B-Cells during Mitogen Stimulation for Memory B-Cell ELISpot Analysis Is Influenced by Size and Composition of the B-Cell Pool
Source: PLoS One. 2014 Jul 22;9(7):e102885. doi: 10.1371/journal.pone.0102885 (PMC4106867; doi:10.1371/journal.pone.0102885)

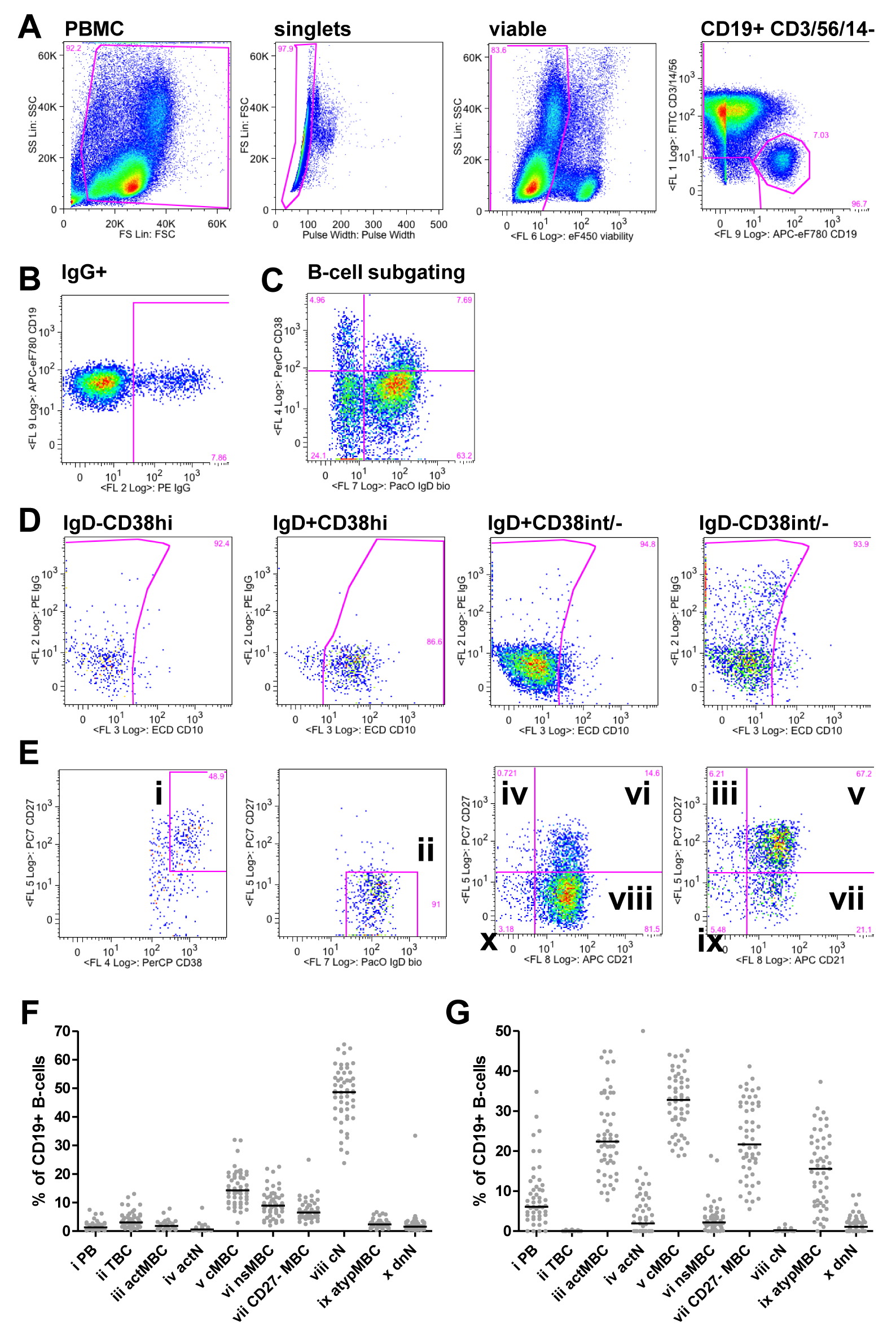

Supplement: Figure S1 — Gating strategy of total and IgG+ B-cells ex vivo . (A) CD19+ B-cell were identified following exclusion of debris, doublets, dead cells and CD3/CD56/CD14-positive cells. (B) shows gating for IgG+ B-cells. This IgG+ gate was later applied to individual B-cell subsets, which were gates as follows: (C) CD19+ B-cells were first subdivided based on IgD and CD38 expression. (D) CD10+ cells were excluded from all B-cell populations except IgD+CD38hi B-cells, which were specifically subgated based on CD10 expression. (E) CD38hi B-cells were divided into CD10−IgD−CD38hiCD27+ plasma blasts (PB, i) and CD10+ IgD+CD38hiCD27− transitional B-cells (TBC, ii). CD38lowCD10− B-cells were subdivided into four pairs of switched/memory (IgD−) and non-switched/naïve (IgD+) B-cell populations: CD21−CD27+ activated MBCs (actMBC, iii) and activated naïve B-cells (actN, iv); CD21+CD27+ classical MBCs (cMBC, v) and non-switched MBCs (nsMBC, vi); CD21+CD27− MBC (CD27− MBC, vii) and classical naïve B-cells (cN, viii); and CD21−CD27− atypical MBCs (atypMBC, ix) and double-negative naïve B-cells (dnN, x). (F) shows the proportions of the ten B-cell subsets of the total ex vivo B-cell pool and (G) the proportion of IgG+ cells within each individual B-cell subset for the baseline samples of all 62 volunteers. (TIF) [file pone.0102885.s001.tif]

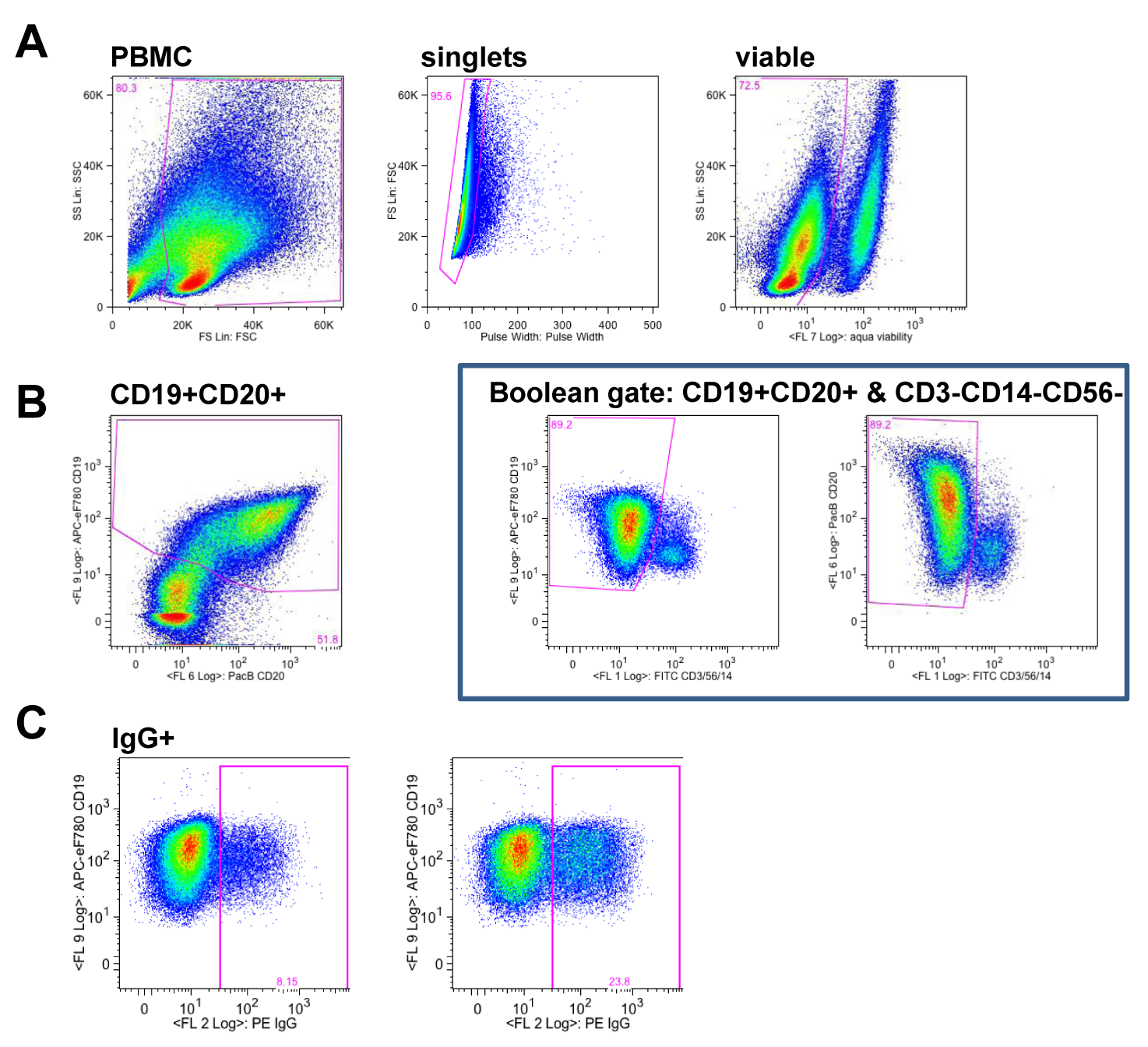

Supplement: Figure S2 — Gating strategy of total and IgG+ B-cells after 5 day mitogen culture. Following (A) exclusion of debris, doublets and dead cells, B-cells were identified by (B) firstly gating on CD19+CD20+ cells and subsequently gating out CD3/CD56/CD14-positive cells (Boolean gating). (C) shows gating for IgG+ B-cells for two donors. (TIF) [file pone.0102885.s002.tif]

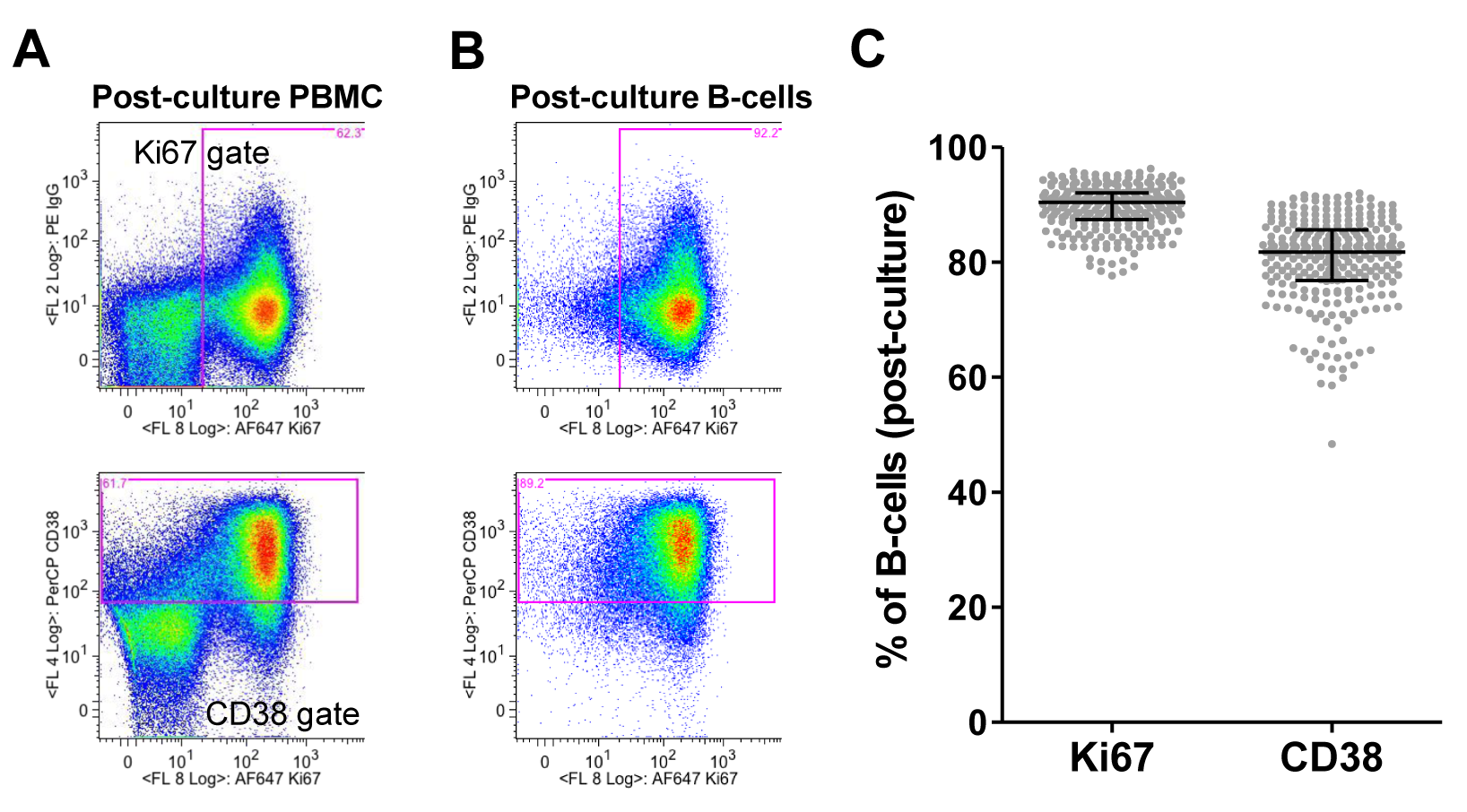

Supplement: Figure S3 — CD38 and Ki67 expression on B-cells after 5 day mitogen culture. (A) Ki67 and CD38 gates were based on negative populations in total post-culture PBMCs (after exclusion of debris, doublets and dead cells). (B) shows Ki67 and CD38 staining on post-culture B-cells. (C) Grey dots show the percentage of Ki67 and CD38 positive cells within post-culture B-cells cultures in all 269 stimulated samples, black lines indicate the median and error bars the interquartile range. (TIF) [file pone.0102885.s003.tif]
