# Supplementary material for: Expansion of IgG+ B-Cells during Mitogen Stimulation for Memory B-Cell ELISpot Analysis Is Influenced by Size and Composition of the B-Cell Pool
Source: PLoS One. 2014 Jul 22;9(7):e102885. doi: 10.1371/journal.pone.0102885 (PMC4106867; doi:10.1371/journal.pone.0102885)
Supplement: Table S1 — B-cells, IgG+ B-cells and MBC subsets in Study A ex vivo samples over time. B-cell proportions and MBC subsets in PBMC samples from Study A volunteers (n = 14 CPS-immunized volunteers and n = 10 controls) were analyzed by flow cytometry. CD19+ B-cell were identified following exclusion of debris, doublets, dead cells and CD3/CD56/CD14-positive. Five MBC populations were identified in the CD38lowCD10− B-cells compartment as followed: IgD−CD21+CD27+ classical MBCs (cMBC), IgD−CD21+CD27− MBC (CD27− MBC), IgD−CD21−CD27+ activated MBCs (actMBC), IgD−CD21−CD27− atypical MBCs (atypMBC) and IgD+CD21+CD27+ non-switched MBCs (nsMBC). (DOC) [file pone.0102885.s004.doc]

**Table S1. B-cells, IgG+ B-cells and MBC subsets in Study A *ex vivo* samples over time**

| **Subset** | **Group** | **I(1)-7** | **C-1** | **C+35** |
| --- | --- | --- | --- | --- |
| **Total B-cells^a^** | Immunized^c^ | 6.0  [3.7-6.6] | 5.4  [3.5-8.9] | 4.5  [3.8-7.4] |
|  | Controls^d^ | 4.8  [3.8-6.0] | 4.7  [4.4-5.7] | 5.0  [3.6-5.5] |
| **IgG+ B-cells^b^** | Immunized | 6.6  [4.4-11.6] | 5.5  [3.9-10.5] | 6.5  [4.5-11.8] |
|  | Controls | 7.5  [4.7-9.7] | 7.1  [4.4-10.3] | 6.4  [3.4-11.0] |
| **cMBC^b^** | Immunized | 13.5  [11.2-19.5] | 12.9  [11.2-16.1] | 12.4  [11.0-18.2] |
|  | Controls | 17.3  [7.7-20.6] | 19.5  [7.7-22.8] | 14.7  [6.1-19.4] |
| **CD27- MBC^b^** | Immunized | 7.7  [5.9-11.4] | 7.2  [5.7-11.3] | 8.1  [6.0-11.0] |
|  | Controls | 6.3  [4.6-7.5] | 5.8  [4.9-7.9] | 5.3  [4.1-7.7] |
| **actMBC^b^** | Immunized | 1.5  [0.8-2.0] | 1.0  [0.7-1.5] | 1.7  [1.1-2.1] |
|  | Controls | 1.7  [0.7-1.3] | 1.2  [1.1-1.8] | 1.8  [0.8-3.5] |
| **atypMBC^b^** | Immunized | 2.4  [1.7-3.1] | 1.9  [1.1-4.1] | 2.3  [1.5-2.9] |
|  | Controls | 2.3  [1.5-3.7] | 2.1  [1.4-2.8] | 2.3  [1.6-3.5] |
| **nsMBC^b^** | Immunized | 8.0  [4.5-11.2] | 8.5  [4.6-11.0] | 8.2  [5.3-11.3] |
|  | Controls | 9.6  [8.8-12.0] | 9.0  [7.1-14.0] | 8.8  [7.8-13.0] |

^a^ Analyzed as percentage of total viable PBMCs; median with interquartile range

^b^ Analyzed as percentage of total B-cells; median with interquartile range

^c^ n= 14 volunteers

^d^ n= 10 volunteers
