# Supplementary material for: Expansion of IgG+ B-Cells during Mitogen Stimulation for Memory B-Cell ELISpot Analysis Is Influenced by Size and Composition of the B-Cell Pool
Source: PLoS One. 2014 Jul 22;9(7):e102885. doi: 10.1371/journal.pone.0102885 (PMC4106867; doi:10.1371/journal.pone.0102885)
Supplement: Table S2 — B-cells, IgG+ B-cells and MBC subsets in Study B ex vivo samples over time. B-cell proportions and MBC subsets in PBMC samples from Study B volunteers (n = 24 CPS-immunized volunteers and n = 5 controls) were analyzed by flow cytometry. CD19+ B-cell were identified following exclusion of debris, doublets, dead cells and CD3/CD56/CD14-positive. Five MBC populations were identified in the CD38lowCD10− B-cells compartment as followed: IgD−CD21+CD27+ classical MBCs (cMBC), IgD−CD21+CD27− MBC (CD27− MBC), IgD−CD21−CD27+ activated MBCs (actMBC), IgD−CD21−CD27− atypical MBCs (atypMBC) and IgD+CD21+CD27+ non-switched MBCs (nsMBC). (DOC) [file pone.0102885.s005.doc]

**Table S2. B-cells, IgG+ B-cells and MBC subsets in Study B *ex vivo* samples over time**

| **Subset** | **Group** | **I(1)-7** | **I(1)+28** | **I(2)+28** | **I(3)+28** | **C-1** | **C+35** | **C+140** |
| --- | --- | --- | --- | --- | --- | --- | --- | --- |
| **Total B-cells^a^** | Immunized^c^ | 7.3  [4.2-8.5] | 5.4  [3.9-7.0] | 6.0  [3.7-6.9] | 5.2  [4.1-7.4] | 6.9  [4.6-9.2] | 7.8  [5.4-10.0] | 7.7  [5.0-9.1] |
|  | Controls^d^ | 7.1  [5.4-9.2] | n.d. | n.d. | n.d. | 7.8  [5.0-8.6] | 7.6  [6.6-9.4] | 6.9  [5.7-9.5] |
| **IgG+ B-cells^b^** | Immunized | 8.0  [6.3-12.7] | 8.1  [6.0-12.1] | 7.9  [6.4-10.0] | 8.4  [6.4-13.0] | 8.2  [5.6-11.5] | 8.1  [6.0-11.9] | 8.3  [5.6-11.5] |
|  | Controls | 7.5  [5.2-11.3] | n.d. | n.d. | n.d. | 6.3  [5.0-12.1] | 7.0  [4.6-10.3] | 6.7  [5.7-11.4] |
| **cMBC^b^** | Immunized | 13.8  [10.1-18.6] | 13.2  [9.4-18.1] | 13.6  [9.7-16.9] | 13.1  [10.0-19.0] | 13.9  [11.1-18.4] | 12.7  [9.9-16.6] | 13.1  [10.2-17.4] |
|  | Controls | 14.9  [12.2-19.7] | n.d. | n.d. | n.d. | 16.2  [11.2-20.0] | 14.2  [11.0-18.3] | 14.5  [11.9-19.0] |
| **CD27- MBC^b^** | Immunized | 6.0  [5.0-8.1] | 5.7  [4.5-8.0] | 5.6  [4.6-7.5] | 5.5  [4.7-7.9] | 5.6  [4.7-8.8] | 6.6  [5.1-8.6] | 5.5  [4.6-9.2] |
|  | Controls | 6.6  [4.6-7.3] | n.d. | n.d. | n.d. | 6.7  [4.7-7.6] | 7.3  [4.6-8.3] | 7.0  [4.5-8.0] |
| **actMBC^b^** | Immunized | 1.9  [1.5-2.5] | 2.2  [1.4-3.2] | 2.3  [1.6-3.6] | 1.9  [1.4-2.7] | 1.6  [1.3-2.7] | 2.0  [1.4-2.4] | 1.5  [1.3-2.1] |
|  | Controls | 2.2  [1.3-4.6] | n.d. | n.d. | n.d. | 2.3  [2.0-2.8] | 2.8  [2.2-2.9] | 2.3  [1.7-2.7] |
| **atypMBC^b^** | Immunized | 2.4  [1.7-3.2] | 2.3  [1.5-3.0] | 2.0  [1.4-3.1] | 1.9  [1.5-3.1] | 1.8  [1.0-2.8] | 1.9  [1.5-3.5] | 1.6  [1.4-3.4] |
|  | Controls | 1.5  [1.3-2.1] | n.d. | n.d. | n.d. | 1.6  [1.0-2.5] | 2.0  [1.3-3.2] | 2.1  [1.5-2.3] |
| **nsMBC^b^** | Immunized | 8.6  [5.6-13.0] | 8.3  [5.5-13.2] | 7.6  [4.8-12.5] | 7.5  [5.2-11.1] | 8.0  [5.2-13.1] | 7.7  [5.7-12.0] | 6.9  [4.9-15.1] |
|  | Controls | 11.7  [9.0-12.7] | n.d. | n.d. | n.d. | 11.1  [6.6-16.7] | 9.6  [6.9-14.5] | 12.8  [7.7-13.6] |

^a^ Analyzed as percentage of total viable PBMCs; median with interquartile range

^b^ Analyzed as percentage of total B-cells; median with interquartile range

^c^ n= 24 volunteers

^d^ n= 5 volunteers
